# The Ministry of Health and the Medical Profession

**Published:** 1919-05-31

**Authors:** 


### The Hospital

The Workers' Newspaper of Administrative Medicine and Institutional Life, Administration, National Insurance and Health.

No. 1721, Vol. LXVI. SATURDAY, MAY 31, 1919. PRICE TWOPENCE

### THE MINISTRY OF HEALTH AND THE MEDICAL PROFESSION.

It has been stated authoritatively that the Government have ready for introduction a Bill which will modify the conditions of medical practice in this country even more markedly than the National Insurance Acts have done already. Meanwhile the various organisations which more or less completely act as collective bargainers for the medical profession—first and foremost the British Medical Association, but also various Royal Colleges and less ancient corporations—have been active in taking part in discussions with the National Insurance Commissioners and others; and it is sincerely to be hoped that the joint result of their labours and of those of Dr. Addison will prove to be a reorganisation satisfactory both to the public and to the doctors. To illustrate the careful attention which the medical profession and the Government departments are paying to the matter, it may suffice to mention that five conferences have been convened by the Health Insurance Commissioners, and that the report of the conclusions reached has recently been circularised officially to every registered practitioner. The main subject of these conferences was, naturally, the administration of the medical services under the Insurance Acts; but larger subjects were also taken under discussion, such, for example, as that of "services for much wider classes of the community" . . . "which must naturally receive early consideration after the establishment of the Ministry of Health."

Until the proposals which Dr. Addison will lay before Parliament are published, speculation as to their nature would be injudicious. But the whole tone of a most remarkable pronouncement which appeared in the *Times* on May 26, and has every appearance of being semi-inspired, or at the least of being a *ballon d'essai* based upon good information, makes it necessary to prepare both the profession and the public for a far-reaching and possibly a highly controversial measure. Bearing in mind the ill-feeling and the disputes caused on the introduction of the Insurance Act, the grave drawbacks which are now admitted to have followed the hasty conception and drafting of that scheme, and to have been the causes of its partial failure to achieve the objects in view, it might be supposed that further experiment along the lines of a State medical service would be marked by more deliberate and less provocative tactics. There is still, it may be hoped, such a prospect; but the *Times* correspondent takes it for granted that a conflict between Dr. Addison and the medical profession is inevitable, and proceeds to browbeat the latter as if the contest were already commenced. He even says that the Government measure is being hurried on, because at present there are numbers of demobilised doctors at a loose end, whom the Government think will be induced to act as strike-breakers if the profession tries to boycott the scheme! "Mr. Addison is evidently determined not to miss his tide," concludes this paragraph. It is sincerely to be hoped that the Machiavellian policy thus attributed to him is a complete misrepresentation of his real intentions; and that the minatory language used in the article referred to is equally foreign to his outlook. If not, if Mr. Addison really contemplates embarking on hurried legislation, calculated to arouse intense opposition and governed more by ephemeral considerations of tactics than by sound statesmanship, then it would indeed seem to be time for the doctors to take to politics in earnest and to support their professional organisations to the utmost of their power.

The promised Bill, according to the authority already quoted, is to be of a "somewhat advanced kind." It is said to be unlikely that it will amount to what is usually understood by a whole-time State Medical Service, but that it may include a system of health centres throughout the country, with hospital and outdoor accommodation, "possibly on the lines suggested by Sir Bertrand Dawson." As the latter's principal suggestions were essentially with a view to a State Medical Service, these hints make it clear that a very comprehensive scheme is to be expected. Still, the proposals evidently do not go so far as to destroy private practice entirely. Until they are tabled, discussion of their purport is impossible; but it is not too early to enter a most emphatic protest against attempts to intimidate the doctors beforehand, and to coerce them by threats into the acceptance of ill-digested schemes which their sense of the public welfare may induce them to oppose. Unless some such intention on the part of the Ministry of Health is shortly to be made manifest, it is difficult to see why this very alarming and offensive forecast of trouble ahead should have been allowed to appear in the most responsible organ of the daily Press. It must also not be forgotten that the public is vitally interested in the scheme, though its opinions may be less vocal than that of the doctors as expressed through the British Medical Association. If another election were imminent, as some observers have lately thought, the temptation to a piece of mere vote-catching might prove irresistible; as to this also there is no more to be said at present, except to hope for the best, in spite of a slightly unpropitious outlook.

